# Rates of Medicare Enrollment Among Dialysis Patients After Implementation of Medicare Payment Reform and the Affordable Care Act Marketplace

**DOI:** 10.1001/jamanetworkopen.2022.32118

**Published:** 2022-09-20

**Authors:** Virginia Wang, Lindsay Zepel, Bradley G. Hammill, Abby Hoffman, Caroline E. Sloan, Matthew L. Maciejewski

**Affiliations:** 1 Department of Population Health Sciences, Duke University School of Medicine, Durham, North Carolina; 2 Department of Medicine, Duke University School of Medicine, Durham, North Carolina; 3 Durham Center of Innovation to Accelerate Discovery and Practice Transformation, Durham Veterans Affairs Health Care System, Durham, North Carolina; 4 Population Health Management Office, Duke Health, Duke University Health System, Durham, North Carolina

## Abstract

**Importance:**

Medicare finances health care for most US patients with end-stage kidney disease (ESKD), regardless of age. The 2011 Medicare prospective payment system (PPS) for dialysis reduced reimbursement for hemodialysis, and the 2014 Patient Protection and Affordable Care Act (ACA) Marketplace increased patient access to new private insurance options, potentially influencing organizations that provide health care, such as hospitals, nursing homes, and dialysis facilities, to adjust their payer mix away from Medicare sources.

**Objective:**

To describe Medicare enrollment trends among patients with incident ESKD in 2006 to 2016.

**Design, Setting, and Participants:**

This retrospective cohort study involved US patients aged 18 to 64 years who were not enrolled in Medicare at dialysis initiation in 2006 to 2016, with 1-year follow-up through 2017. Data analysis was conducted April 2021 to June 2022.

**Exposures:**

The exposure of interest was a 3-category indicator of time, whether patients initiated dialysis before policies were enacted (2006-2010), in the first years of the Medicare ESKD PPS (2011-2013), or during the Medicare ESKD PPS and implementation of the ACA Marketplace (2014-2016).

**Main Outcomes and Measures:**

Patient-level Medicare enrollment through the first year of dialysis. Logistic regression and Cox models were used to examine associations of time, patient characteristics, and Medicare enrollment, adjusting for patient demographic, clinical, and market-level characteristics.

**Results:**

Of 335 157 patients aged 18 to 64 years with ESKD not actively enrolled in Medicare when they initiated dialysis in 2006 to 2016, the mean (SD) age was 49.9 (10.8) years, 198 164 (59.1%) were men, 188 290 (56.2%) were White, and 313 622 (93.6%) received in-center hemodialysis. New Medicare enrollment was higher in 2006 to 2010 (110 582 patients [73.1%]) than after the Medicare ESKD PPS and ACA Marketplace in 2014 to 2016 (55 382 patients [58.5%]). In adjusted analyses, declining Medicare enrollment was associated with implementation of 2011 Medicare ESKD PPS and 2014 ACA policies and was disproportionately lower among younger, racially minoritized, and ethnically Hispanic patients.

**Conclusions and Relevance:**

There was declining Medicare enrollment among new dialysis patients associated with the 2011 Medicare ESKD PPS and 2014 ACA Marketplace that raise concerns about benefits and harms to patients and payers and continued disparities in kidney care. As the dialysis payer mix moves toward higher proportions of patients not covered by Medicare, it will be important to understand the implications for health care system and patient outcomes.

## Key Points

**Question:**

Did Medicare enrollment decline among new dialysis patients after implementation the 2011 Medicare dialysis prospective payment system and the 2014 Patient Protection and Affordable Care Act (ACA) Marketplace?

**Findings:**

In this cohort study of nonelderly patients initiating dialysis from 2006 to 2016, Medicare enrollment declined, especially after implementation of the 2014 ACA Marketplace. Moreover, Medicare enrollment was disproportionately lower among younger, racially minoritized, and ethnically Hispanic patients.

**Meaning:**

As dialysis patient payer mix moves toward higher proportions of patients not covered by Medicare, it will be important to continue monitoring these trends and understand the implications for health care system and patient outcomes.

## Teaser

This cohort study describes Medicare enrollment trends among patients with incident end-stage kidney disease in 2006 to 2016.

## Introduction

Since the 1972 legislative passage of near-universal health coverage for patients with end-stage kidney disease (ESKD) younger than 65 years, an overwhelming majority (up to 92%) of US individuals with kidney failure have been covered by Medicare.^1^ A smaller proportion of patients with ESKD have private insurance or lack coverage. Organizations that provide health care, such as hospitals, nursing homes, and dialysis facilities, are acutely sensitive to changes in Medicare reimbursement, which is lower than private payer rates.^2,3,4,5^ When Medicare reimbursements are deemed too low, dialysis providers may care for fewer Medicare patients or pursue other revenue (payer) sources. Policy changes in the past decade may have induced dialysis facilities to shift their payer mix away from Medicare.

In 2011, Medicare established a new prospective payment system (PPS) for dialysis services that resulted in decreased payment for some dialysis drugs and overall revenues for dialysis care and, thus, increased the difference between Medicare and private insurance. Then in 2014, the Patient Protection and Affordable Care Act (ACA) effectively increased access to private insurance for patients with ESKD by creating private insurance marketplaces, expanding special enrollment periods, and prohibiting coverage denials to patients with preexisting conditions.^6^ Charitable premium assistance programs, which are funded in part by dialysis provider organizations, have enabled some patients with ESKD to obtain or maintain private insurance (eg, employer group insurance, COBRA [Consolidated Omnibus Budget Reconciliation Act] coverage, and ACA guaranteed issue rules).^2,6,7^ In combination, these policies and programs have raised concerns about alleged efforts by dialysis facilities to optimize revenues by increasing their proportion of privately insured patients and decreasing their reliance on Medicare.

In prior research,^8^ dialysis facilities reported that Medicare enrollment among their patients declined after the 2011 dialysis PPS and 2014 ACA Marketplace were implemented, from an average of 89% of patients per dialysis facility in 2005 to an average of 65% in 2016. The share of patients who were enrolled in non-Medicare coverage simultaneously increased. These estimates reflect Medicare enrollment on patients with prevalent (ie, existing) ESKD, which may mask important enrollment trends among the subset of patients who are not yet enrolled and eligible for Medicare at the onset of kidney failure.

This study describes patient-level trends in Medicare enrollment for patients with newly diagnosed ESKD in 2006 to 2016 who had to decide whether to enroll in Medicare and examines whether the 2011 and 2014 policy changes changed their decisions. Findings from this work can inform whether these policy changes have unintended effects on enrollment among dialysis patients newly eligible for Medicare.

## Methods

### Study Design, Population, and Data Sources

The study protocol and consent waiver were approved by Duke University; consent was waived because the data were secondary administrative data, in accordance with 45 CFR §46. This study followed the Strengthening the Reporting of Observational Studies in Epidemiology (STROBE) reporting guideline.

We conducted a retrospective cohort study of all US patients aged 18 to 64 years (below the age threshold for Medicare enrollment) with incident ESKD initiating dialysis treatment between 2006 and 2016, with 1-year follow-up through 2017. The prepolicy period comprised years 2006 to 2010. The first postpolicy period reflected the years during which the Medicare PPS was in effect but the ACA Marketplace was not (2011-2013). The second postpolicy period comprised the years during which both the Medicare PPS and the ACA Marketplace were in effect (2014-2016). Patients were excluded if they had missing demographic information, had prevalent ESKD, died within 90 days of initiating dialysis, received a kidney transplant within 90 days of initiating dialysis, recovered kidney function within 180 days of initiating dialysis, had no record of modality type, or had missing or invalid zip codes. We excluded patients with active Medicare enrollment at dialysis initiation because we wanted to assess new Medicare enrollment among patients who were eligible for Medicare at ESKD onset (eFigure 1 in the Supplement).

The US Renal Data System (USRDS)^9^ was the primary source of data. Medicare enrollment and non-Medicare status were ascertained from the longitudinal USRDS Payer History file. Patient demographic and clinical characteristics were obtained from USRDS Patient and Medical Evidence Report (Centers for Medicare & Medicaid Services form 2728) files, which is completed whenever any patient with ESKD begins care at a dialysis facility (regardless of payer source). Dialysis modality data were from the USRDS Treatment History file. Demographic statistics from the Area Health Resource File were converted and then aggregated to generate market-level (hospital service areas)^10^ statistics for each year of the study period.

### Measurement

The outcome of interest was Medicare enrollment status 1 year after dialysis initiation (eg, first day of dialysis). Medicare enrollment was defined as being actively enrolled in Medicare Advantage or traditional Medicare (as either primary or secondary payer). This was assessed by the 2 decision points in which patients were eligible for and decided whether to enroll in Medicare: on day 1 of dialysis (initiation) or days 2 to 365 of dialysis. On day 1 of dialysis (initiation), patients without active enrollment were either pending Medicare enrollment or had other coverage (eg, Medicaid, private, or none). Patients with pending Medicare enrollment on day 1 of dialysis applied for Medicare in the time between the onset of ESKD and their first day of dialysis treatment. Most patients pending new Medicare enrollment are required to undergo a 90-day waiting period (but the waiting period is waived for patients initiating home dialysis), and are thereby actively enrolled in Medicare by the end of their first year undergoing dialysis (eFigure 2 in the Supplement). Historically, most patients with new ESKD make decisions about Medicare enrollment at this time. Patients deciding to enroll in Medicare on days 2 to 365 of dialysis may have other sources of coverage and do not apply for Medicare at dialysis initiation for various reasons (eg, emergent dialysis or logistical transition to dialysis routines) but still have the option to enroll in Medicare. The subset of patients with other coverage were the focus of the days 2 to 365 analysis, and those with pending Medicare enrollment were excluded. Thus, we assessed Medicare enrollment status on the first day of dialysis through 1 year after dialysis initiation.

The explanatory variable of interest was a 3-category variable of time, defined by whether dialysis initiation occurred before policies of interest were implemented (2006-2010), in the first years of the Medicare PPS (2011-2013), or during the Medicare PPS and the ACA Marketplace (2014-2016). Medicare enrollment was also assessed by patient age (eg, ages 18-64 years are not commonly enrolled in Medicare without ESKD) and reported race (eg, Black, White, or other [ie, American Indian/Alaskan Native, Asian, Native Hawaiian, or other Pacific Islander]) and Hispanic ethnicity. Race and ethnicity were evaluated as social constructs and as demographic indicators to identify potential patient subgroups associated with shifts away from Medicare enrollment (eg, access to Medicare). Other characteristics that may be associated with Medicare enrollment were included to account for differences in the study population’s composition across the 3 time periods. These included patient characteristics at the start of dialysis, such as sex, US region, employment status, urban residence, cause of ESKD, treatment modality (in-center hemodialysis or home-based hemodialysis or peritoneal dialysis), receipt of pre-ESKD nephrology care, baseline kidney function,^11^ baseline body mass index, and comorbid conditions. Patients’ regional characteristics included dialysis facility composition (eg, proportion of freestanding, for-profit, chain-affiliated, and urban facilities), general population demographics (eg, proportion of urban residents, per capita income in the general population), and dialysis facility competition,^12,13,14^ based on the number of dialysis patients unique to each facility.^15^

### Statistical Analysis

We examined enrollment trends among patients who made Medicare enrollment decisions on day 1 of dialysis (those pending Medicare enrollment on day 1) and days 2 to 365 of dialysis (for those with other coverage) (eFigure 1 and eFigure 2 in the Supplement). For patients making decisions about Medicare enrollment on days 2 to 365 of dialysis, the 1-year cumulative incidence of Medicare enrollment was determined using the cumulative incidence function. Follow-up time started at dialysis initiation and patients were censored at the earliest of (1) 1 year after dialysis initiation or (2) end of data availability (December 31, 2017). Death and kidney transplant were treated as competing events.

In adjusted analyses of Medicare enrollment at the end of the first year of dialysis, a logistic regression model was used to examine associations between the enrollment decision on the day 1 of dialysis and patient demographic, clinical, and hospital service areas market characteristics. In the subset of patients with other coverage at dialysis initiation deciding to enroll in Medicare on days 2 to 365 of dialysis, a Cox model was used to assess the association between Medicare enrollment and patient demographic, clinical, and market characteristics. Analysis was conducted from April 2021 to June 2022. Data were analyzed using SAS statistical software version 9.4 (SAS Institute).

## Results

### Patient Characteristics

Of the cohort’s 335 157 patients who were not actively enrolled in Medicare when initiating dialysis in 2006 to 2016, the mean (SD) age was 49.9 (10.8) years, 198 164 (59.1%) were men, 188 290 (56.2%) were White, 278 446 (83.3%) resided in urban areas, 187 119 (55.8%) had received nephrology care before the onset of ESKD, and 313 622 (93.6%) underwent in-center hemodialysis (Table 1). A smaller proportion were Black (121 279 patients [36.2%]), were of Hispanic ethnicity (63 382 patients [18.9%]), identified as other races (25 588 patients [7.6%]), and had either full or partial employment (81 548 patients [24.3%]). New dialysis patients lived in regions with high concentrations of dialysis facilities that were freestanding (vs hospital-based) (mean [SD], 91.5% [17.8%]), for-profit owned (mean [SD], 84.8% [24.1%]), and affiliated with chain organizations (mean [SD], 86.5% [21.7%]). These demographic trends were stable across the 3 policy periods and also similar across patients determining Medicare enrollment at day 1 and days 2 to 365 of dialysis initiation (Table 1 and eTable in the Supplement).

**Table 1.** Characteristics of Patients Aged 18 to 64 Years With No Active Medicare Enrollment at Dialysis Initiation, 2006 to 2016

Patient characteristics, at dialysis initiation	Patients, No. (%)			
	Overall (N = 335 157)	2006-2010 (n = 151 287)	2011-2013 (n = 89 158)	2014-2016 (n = 94 712)
Age, mean (SD), y	49.9 (10.8)	49.7 (10.8)	50.1 (10.9)	50.0 (10.8)
18-44	92 089 (27.5)	42 236 (27.9)	23 889 (26.8)	25 964 (27.4)
45-54	103 769 (31.0)	47 695 (31.5)	27 435 (30.8)	28 639 (30.2)
55-64	139 299 (41.6)	61 356 (40.6)	37 834 (42.4)	40 109 (42.3)
Sex				
Male	198 164 (59.1)	87 694 (58.0)	53 175 (59.6)	57 295 (60.5)
Female	136 993 (40.9)	63 593 (42.0)	35 983 (40.4)	37 417 (39.5)
Race				
Black	121 279 (36.2)	57 660 (38.1)	31 578 (35.4)	32 041 (33.8)
White	188 290 (56.2)	82 667 (54.6)	50 894 (57.1)	54 729 (57.8)
Other^a^	25 588 (7.6)	10 960 (7.2)	6686 (7.5)	7942 (8.4)
Hispanic ethnicity	63 382 (18.9)	27 567 (18.2)	17 512 (19.6)	18 303 (19.3)
Employed full-time or part-time	81 548 (24.3)	36 320 (24.0)	20 633 (23.1)	24 595 (26.0)
Urban residential status	278 446 (83.3)	125 719 (83.3)	74 155 (83.5)	78 572 (83.3)
Region				
South	143 357 (42.8)	64 797 (42.8)	37 912 (42.5)	40 648 (42.9)
Midwest	63 330 (18.9)	29 068 (19.2)	16 683 (18.7)	17 579 (18.6)
Northeast	53 110 (15.8)	24 263 (16.0)	14 131 (15.8)	14 716 (15.5)
West	75 360 (22.5)	33 159 (21.9)	20 432 (22.9)	21 769 (23.0)
Dialysis modality				
In-center hemodialysis	313 622 (93.6)	144 527 (95.5)	83 117 (93.2)	85 978 (90.8)
Home hemodialysis	629 (0.2)	203 (0.1)	206 (0.2)	220 (0.2)
Peritoneal dialysis	20 906 (6.2)	6557 (4.3)	5835 (6.5)	8514 (9.0)
Cause of ESKD				
Diabetes	157 055 (46.9)	70 168 (46.4)	41 736 (46.8)	45 151 (47.7)
Hypertension	89 157 (26.6)	38 076 (25.2)	24 478 (27.5)	26 603 (28.1)
Glomerulonephritis	40 077 (12.0)	19 140 (12.7)	10 546 (11.8)	10 391 (11.0)
Other	40 466 (12.1)	19 076 (12.6)	10 179 (11.4)	11 211 (11.8)
Unknown	8402 (2.5)	4827 (3.2)	2219 (2.5)	1356 (1.4)
Comorbidities^b^				
Hypertension	296 547 (88.5)	133 187 (88.0)	79 332 (89.0)	84 028 (88.7)
Diabetes	181 699 (54.2)	80 084 (52.9)	48 847 (54.8)	52 768 (55.7)
Congestive heart failure	71 418 (21.3)	34 263 (22.6)	18 549 (20.8)	18 606 (19.6)
Atherosclerotic heart disease	32 756 (9.8)	17 310 (11.4)	8496 (9.5)	6950 (7.3)
Peripheral vascular disease	24 272 (7.2)	12 202 (8.1)	6220 (7.0)	5850 (6.2)
Pre-ESKD nephrology care				
Yes	187 119 (55.8)	81 380 (53.8)	49 833 (55.9)	55 906 (59.0)
No	107 070 (31.9)	52 596 (34.8)	28 452 (31.9)	26 022 (27.5)
Unknown	40 968 (12.2)	17 311 (11.4)	10 873 (12.2)	12 784 (13.5)
Body mass index, mean (SD)^c^	30.3 (8.4)	30.1 (8.4)	30.5 (8.5)	30.5 (8.4)
eGFR, mean (SD), mL/min/1.73 m^2^^d^	10.2 (11.8)	10.5 (12.7)	10.3 (11.8)	9.7 (10.1)
Market characteristics (hospital service area), mean (SD)				
Freestanding facilities, %	91.5 (17.8)	89.7 (19.6)	92.1 (17.0)	93.7 (14.8)
For-profit owned, %	84.8 (24.1)	82.7 (25.8)	85.5 (23.4)	87.5 (21.5)
Chain affiliated, %	86.5 (21.7)	83.9 (23.8)	86.9 (21.1)	90.3 (17.7)
Urban facility location, %	85.7 (33.0)	85.8 (32.9)	85.8 (32.9)	85.4 (33.3)
Dialysis market competition^e^	39.4 (34.7)	38.8 (34.6)	39.5 (34.7)	40.3 (34.9)
Urban general population, %	83.1 (20.8)	83.1 (20.9)	83.2 (20.8)	83.1 (20.8)
Per capita annual income, median (IQR), $	40 461 (34 588-47 001)	37 218 (31 997-43 172)	41 657 (35 979-47 068)	44 516 (38 726-52 783)
Insurance status at day 1 of dialysis initiation				
Pending Medicare enrollment	205 671 (61.4)	101 821 (67.3)	55 067 (61.8)	48 783 (51.5)
Other coverage	129 486 (38.6)	49 466 (32.7)	34 091 (38.2)	45 929 (48.5)

Abbreviations: ESKD, end-stage kidney disease; eGFR, estimated glomerular filtration rate.

^a^ Other race included American Indian/Alaskan Native, Asian, Native Hawaiian, or other Pacific Islander.

^b^ Other comorbidities include chronic obstructive pulmonary disease, cerebrovascular disease, inability to ambulate, inability to transfer, other cardiac disease, cancer, drug dependence, tobacco use.

^c^ Body mass index is calculated as weight in kilograms divided by height in meters squared.

^d^ Calculation of eGFR is based on the Chronic Kidney Disease Epidemiology Collaboration formula.

^e^ Measured using the Herfindahl-Hirschman Index of dialysis market competition, which is equal to the sum of the square of each dialysis facility’s market share, based on the number of dialysis patients unique to each facility. Index values range from 0 to 100, where a value of 0 reflects unconcentrated, competitive markets and values approaching 100 characterize concentrated, monopolistic markets.

### Trends in Medicare Enrollment

Among patients not actively enrolled in Medicare at dialysis initiation in 2006 to 2016, 67.8% (227 208 patients) were enrolled in Medicare by the end of their first year undergoing dialysis. New Medicare enrollment was higher in 2006 to 2010 than in 2014 to 2016 after implementation of the ACA (110 582 patients [73.1%] vs 55 382 patients [58.5%]) (Figure and eFigure 3 in the Supplement). Much of this decline was associated with reductions in the proportion of patients who had applied and were pending Medicare enrollment on day 1 of dialysis (67.3% [101 821 patients] in 2006-2010 to 51.5% [48 783 patients] in 2014-2016). The proportion of patients who determined Medicare enrollment during days 2 to 365 of dialysis (eg, those with other coverage at dialysis initiation) also declined (17.7% [8768 patients] in 2006-2010 to 14.4% [6602 patients] in 2014-2016).

**Figure.**
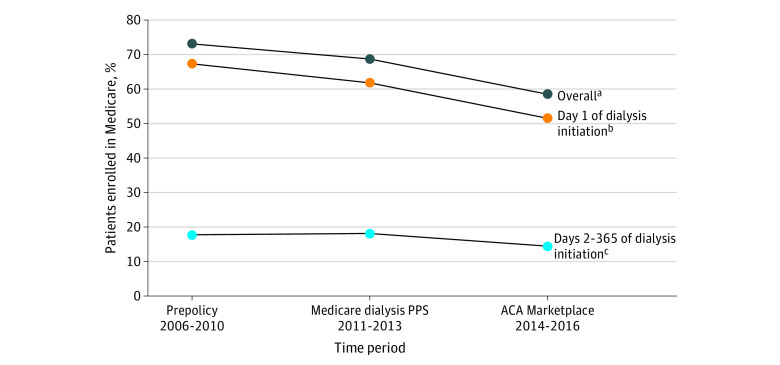
Medicare Enrollment 1 Year After Dialysis Initiation, by Timing of Medicare Enrollment Decision (N = 335 157) Source: Authors’ analysis of data from the US Renal Data System. ACA indicates Patient Protection and Affordable Care Act; PPS, prospective payment system. ^a^Rates reported in the overall sample reflect the proportion of patients at-risk for Medicare enrollment who decided to enroll in Medicare by the end of the first year of dialysis initiation. Among patients with newly diagnosed end-stage kidney disease (ESKD) in 2006 to 2010 not enrolled in Medicare at dialysis initiation, 73.1% decided to and were enrolled in Medicare by the end of the first year on dialysis. This rate declined to 58.5% in 2014 to 2016. ^b^Rates reported in the day 1 of dialysis initiation subgroup reflects the proportion of patients at-risk for Medicare enrollment who decided to enroll in Medicare on day 1. Among patients not enrolled in Medicare at dialysis initiation in 2006 to 2010, 67.3% decided to enroll in Medicare on day 1. The rate declined to 51.5% in 2014 to 2016. ^c^Rates reported in the days 2 to 365 of dialysis initiation subgroup reflect the 1-year cumulative incidence of Medicare enrollment among patients who did not make the decision to enroll in Medicare on day 1 and had decided between days 2 and 365. Of patients with newly diagnosed ESKD in 2006 to 2010 who did not make the decision to enroll in Medicare on day 1, 17.7% did so between days 2 to 365. This declined to 14.4% in 2014 to 2016.

In adjusted analysis, there were lower and decreasing odds of Medicare enrollment among eligible patients on day 1 of dialysis (ie, pending Medicare) after the 2011 to 2013 Medicare ESKD PPS period compared with the 2006 to 2010 prepolicy period (odds ratio [OR], 0.82; 95% CI, 0.81-0.84), between the 2011 to 2013 Medicare ESKD PPS and the 2014 to 2016 ACA Marketplace periods (OR, 0.67; 95% CI, 0.67-0.70), and the overall postpolicy periods of both Medicare ESKD PPS and ACA Marketplace than the 2006 to 2010 prepolicy period (OR, 0.56; 95% CI, 0.55-0.57) (Table 2). Patients aged 45 to 55 years had higher odds of pending Medicare enrollment than did patients aged 55 to 64 years (OR, 1.04; 95% CI, 1.03-1.06). However, pending Medicare enrollment at dialysis initiation was lower among new dialysis patients who were Black vs those who were White (OR, 0.88; 95% CI, 0.86-0.90) and other races (hazard ratio [HR], 0.85; 95% CI, 0.82-0.88). Regionally, lower odds of pending Medicare enrollment were observed in the Midwest (OR, 0.93; 95% CI, 0.61-0.64), Northeast (OR, 0.47; 95% CI, 0.46-0.48), and West (OR, 0.60; 95% CI, 0.58-0.61) vs the US South.

**Table 2.** Adjusted Results: Medicare Enrollment 1 Year After Dialysis Initiation, by Time of Medicare Enrollment Decision, 2006-2016^a^

Characteristic	Day 1 of dialysis initiation, OR (95% CI) (n = 335 157)	Days 2-365 of dialysis, HR (95% CI) (n = 129 486)
Year of dialysis initiation		
2011-2013 vs 2006-2010	0.82 (0.81-0.84)	0.98 (0.95-1.02)
2014-2016 vs 2011-2013	0.68 (0.67-0.70)	0.76 (0.73-0.78)
2014-2016 vs 2006-2010	0.56 (0.55-0.57)	0.74 (0.72-0.77)
Age at dialysis initiation, y		
18-44	1.00 (0.98-1.02)	0.61 (0.59-0.64)
45-54	1.04 (1.03-1.06)	0.69 (0.67-0.71)
55-64	1 [Reference]	1 [Reference]
Sex		
Male	1.17 (1.15-1.18)	1.12 (1.09-1.16)
Female	1 [Reference]	1 [Reference]
Race		
Black	0.88 (0.86-0.90)	0.81 (0.79-0.84)
White	1 [Reference]	1 [Reference]
Other^b^	0.85 (0.82-0.88)	0.71 (0.67-0.75)
Ethnicity		
Hispanic	1.00 (0.98-1.02)	0.78 (0.75-0.81)
Non-Hispanic	1 [Reference]	1 [Reference]
Cause of ESKD		
Diabetes	1 [Reference]	1 [Reference]
Hypertension	0.98 (0.95-1.00)	0.93 (0.89-0.97)
Glomerulonephritis	1.00 (0.97-1.03)	1.04 (0.98-1.10)
Other	0.87 (0.85-0.90)	0.94 (0.89-0.99)
Unknown	0.87 (0.83-0.92)	0.75 (0.67-0.83)
Employment status		
Full-time or part-time	1.01 (0.99-1.03)	1.03 (0.99-1.06)
Unemployed	1 [Reference]	1 [Reference]
Body mass index, per 5^c^	1.02 (1.01-1.02)	1.02 (1.01-1.02)
Pre-ESKD care		
No	1 [Reference]	1 [Reference]
Yes	0.97 (0.95-0.98)	1.14 (1.10-1.18)
Unknown	0.81 (0.79-0.83)	1.00 (0.95-1.05)
Region		
South	1 [Reference]	1 [Reference]
Midwest	0.63 (0.61-0.64)	0.93 (0.90-0.97)
Northeast	0.47 (0.46-0.48)	0.79 (0.75-0.82)
West	0.60 (0.58-0.61)	0.81 (0.78-0.84)
Urban	0.96 (0.91-1.00)	1.04 (0.95-1.15)
Nonurban	1 [Reference]	1 [Reference]
Dialysis modality on day of dialysis initiation		
Hemodialysis	1 [Reference]	1 [Reference]
Home dialysis	0.41 (0.34-0.48)	1.16 (0.92-1.46)
Peritoneal dialysis	0.31 (0.30-0.32)	1.00 (0.96-1.05)
Estimated glomerular filtration rate	1.00 (1.00-1.00)	1.00 (1.00-1.00)
Comorbidities		
No	1 [Reference]	1 [Reference]
Hypertension	1.15 (1.13-1.18)	1.02 (0.98-1.07)
Diabetes	1.00 (0.98-1.02)	1.03 (0.99-1.08)
Congestive heart failure	0.97 (0.95-0.99)	1.07 (1.03-1.11)
Atherosclerotic heart disease	1.02 (0.99-1.05)	1.10 (1.05-1.15)
Peripheral vascular disease	0.95 (0.92-0.98)	0.99 (0.94-1.05)
Chronic obstructive pulmonary disease	0.82 (0.79-0.85)	0.89 (0.84-0.96)
Cerebrovascular disease or transient ischemic attack	0.91 (0.88-0.94)	1.04 (0.98-1.10)
Cancer	0.88 (0.84-0.91)	0.98 (0.91-1.05)
Drug dependence	0.57 (0.54-0.60)	0.54 (0.48-0.60)
Tobacco use	0.99 (0.96-1.01)	0.84 (0.79-0.88)
Inability to ambulate	0.61 (0.58-0.65)	0.77 (0.70-0.85)
Inability to transfer	0.81 (0.75-0.87)	0.87 (0.75-1.00)
Market characteristics (hospital service area), per 10		
Freestanding facilities, %	1.00 (0.99-1.01)	1.00 (0.99-1.01)
For-profit owned, %	0.98 (0.97-0.98)	1.00 (0.99-1.01)
Chain affiliated, %	1.00 (1.00-1.01)	1.01 (1.00-1.02)
Urban location, %	1.01 (1.00-1.01)	1.00 (0.99-1.01)
Dialysis market competition	1.02 (1.02-1.02)	1.01 (1.01-1.02)
Urban general population, %	0.96 (0.95-0.96)	1.01 (1.00-1.02)
Per capita annual income, per $10 000	0.91 (0.90-0.92)	0.99 (0.98-1.01)

Abbreviations: ESKD, end-stage kidney disease; HR, hazard ratio; OR, odd ratio.

^a^ The outcome was modeled using logistic regression in the day 1 of dialysis initiation analysis (reporting ORs) and Cox regression in the days 2 to 365 of dialysis analysis (reporting HRs).

^b^ Other race included American Indian/Alaskan Native, Asian, Native Hawaiian, or other Pacific Islander.

^c^ Body mass index is calculated as weight in kilograms divided by height in meters squared.

Among the 129 486 patients with no active or pending Medicare deciding to enroll in Medicare during days 2 to 365 of dialysis, Medicare enrollment was not associated with the 2011 Medicare dialysis PPS (HR, 0.98; 95% CI, 0.95-1.02) but was negatively associated with both the 2011 to 2013 Medicare ESKD PPS period compared with the 2014 ACA Marketplace (HR, 0.76; 95% CI, 0.73-0.78) and the 2014 to 2016 Medicare ESKD PPS and ACA Marketplace period compared with the 2006 to 2010 (HR, 0.74; 95% CI, 0.72-0.77) (Table 2). Medicare enrollment through the first year of dialysis was lower for patients who were Black vs those who were White (HR, 0.81; 95% CI, 0.79-0.84) or other races (HR, 0.71; 95% CI, 0.67-0.75) and for those who were ethnically Hispanic vs non-Hispanic (HR, 0.78; 95% CI, 0.75-0.81) (Table 2). Over the entire study period, Medicare enrollment was less likely among younger patients, aged 18 to 44 years (HR, 0.61; 95% CI, 0.59-0.64) and aged 45 to 55 years (HR, 0.69; 95% CI, 0.67-0.71), than among those aged 55 to 64 years (Table 2). Lower hazards of Medicare enrollment were observed in the Midwest (HR, 0.93; 95% CI, 0.90-0.97), Northeast (HR, 0.79; 95% CI, 0.75-0.82), and West (HR, 0.81; 95% CI, 0.78-0.84) than in the South.

## Discussion

This cohort study found that between 2006 and 2016, fewer patients aged 18 to 64 years with incident ESKD enrolled in Medicare. The 2011 Medicare PPS was associated with lower pending Medicare enrollment among patients at dialysis initiation. ACA provisions in 2014, which barred plans from coverage denials due to preexisting conditions and relaxing special enrollment periods, expanded access to private insurance for patients with ESKD.^6^ The time period in which these provisions were enacted was associated with steeper decreases in Medicare enrollment among those newly eligible for Medicare in the first year of dialysis. Together, the findings suggest an additive effect on Medicare enrollment over time.

These results augment prior research^8^ reporting that dialysis facilities’ share of Medicare enrolled patients with prevalent ESKD declined after 2011 and during a period of expansion of non-Medicare, private sources of coverage. The associated trends shown in this analysis of patients with incident ESKD newly eligible to enroll in Medicare’s ESKD program lends credence to allegations of patients being encouraged to maintain private insurance, which reimburses dialysis treatments at substantially higher rates than Medicare.^2,3,4^ Other dynamics of the dialysis industry—such as growth in consolidation, private equity ownership, and kickbacks—may also underlie the behavior of organizations that provide health care and changes in ESKD payer mix but were not examined here. Organizations such as hospitals, nursing homes, and dialysis facilities commonly help patients navigate and identify sources of payment coverage for dialysis. Policy makers are concerned that charitable premium assistance programs play an outsize role in helping patients maintain commercial insurance. However, these programs are partly funded by dialysis organizations,^2,6,7^ presenting a potential conflict of interest. To determine whether patients are increasingly using private insurance with reimbursements rates that are advantageous to these organizations, additional data are needed to discern different types of non-Medicare coverage, which may include Medicaid, Veterans Affairs, and private insurance sources.

Interestingly, we found that the overall decline in Medicare ESKD enrollment was largely associated with a reduction in the proportion of patients who applied for Medicare before or on the day of their first dialysis treatment. Patients may have considerable difficulty making decisions about financing, coverage, and benefits at a particularly disruptive time of transition to dialysis (eg, ESKD diagnosis, education, and preparation for treatment). Insurance enrollment pathways for patients may differ markedly, as those who applied for Medicare by day 1 of dialysis initiation could be exposed to different supports and resources for insurance coverage information (eg, earlier preparation and time to explore financial resources for ESKD care or assistance by social workers at hospitals and dialysis facilities) than those who apply after starting dialysis. For new dialysis patients who have not yet determined eligibility and process for enrolling in Medicare by their first day of dialysis treatment, dialysis providers may have an outsize influence on their decisions. It is also possible that declining Medicare enrollment may be associated with demographic changes among patients with ESKD, such as increasing employment and access to insurance coverage. Further examination of these differences and their associations with patient insurance coverage decisions will have important implications on insurance enrollment and coverage policies.

The shift in payer mix raises concerns about benefits and harms to patients and payers. To date, there is no evidence that increased non-Medicare coverage is associated with patient harm. Lower enrollment in Medicare may differentially affect patients and society. Patients with private insurance may have better outcomes than patients enrolled in Medicare (eg, lower out-of-pocket cost-sharing under charitable premium assistance programs^6^ or better anemia management^16^). Patients may not be able to afford Medicare Part B premiums, the Medicare fee-for-service 20% cost-share for treatment, or have access to Medigap plans that charitable assistance program funding is suited to address. However, patients who receive premium assistance for private insurance are masked from the full costs of care. In contrast, growing non-Medicare coverage for dialysis services has different implications for patients, states, the federal government, and taxpayers. Declines in Medicare enrollment and higher reimbursements from private insurance suggest higher societal costs that affect health systems, insurers, and overall consumer premiums (not limited to patients with ESKD).^7^ Proposed policy requiring dialysis facilities to disclose participation in third-party premium programs may curtail profits and overall costs.^2,6^ In addition, acquisition of private coverage through premium assistance may prevent patients’ timely access to kidney transplant. Since participation in premium assistance programs is conditional on undergoing dialysis, patients are unable to effectively demonstrate continuous coverage through posttransplantation (required for clinical monitoring and immunosuppressant medications).^3^ Finally, our findings also illuminate a troubling trend in disproportionately lower Medicare enrollment among racially minoritized patients with ESKD. Considering existing racial and socioeconomic disparities in kidney disease detection and clinical management before the end stage of disease, our findings may indicate yet another disparity among patients with kidney disease: access to Medicare. The lower Medicare enrollment we found among Black and other racially minoritized patients may exacerbate racial and socioeconomic disparities in access to optimal dialysis care and downstream health care disparities in transplantation.^17,18,19,20^ It will be important for future research to monitor these trends and downstream effects on kidney care and outcomes for policy makers to consider.

### Limitations

This study has several limitations. First, we are unable to accurately determine the type of insurance in which non-Medicare patients were enrolled (eg, Medicaid, Veterans Affairs, or private) or patients’ specific insurance coverage and relied on the USRDS longitudinal records of Medicare enrollment status. Second, the analysis reflects the initial years of post-ACA enrollment in Medicare. Although we expect trends to persist in the longer term, policy initiatives occurring after our observation period further affect insurance coverage for patients with ESKD. Growth in patient enrollment in Medicare Advantage (vs traditional Medicare fee-for-service),^21^ the rollout of value-based payment and care models for kidney disease,^22,23,24^ requirements for organizations that provide health care to disclose third-party payments,^2^ and judicial rulings on employer-group benefits coverage for dialysis services^25,26^ have variable influence in ESKD payer mix trends, and outcomes and will be important to further investigate. Third, despite attempts to adjust for factors to minimize bias, unmeasured confounders may exist and causality cannot be proven.

## Conclusions

This cohort study found a notable decline in Medicare enrollment among patients with new ESKD onset between 2006 and 2016, in the years after enactment of Medicare payment reform and the ACA. Reductions in Medicare enrollment were disproportionately observed among racially minoritized patients with ESKD. Policy makers and researchers should monitor these trends and their implications on health system and patient outcomes.

## Notes

Supplement. **eFigure 1.** Patient Sampling Frame **eFigure 2.** Sample: Patients With Incident End-Stage Kidney Disease, by Insurance Status at Dialysis Initiation 2006-2016 **eTable.** Characteristics of Patients Age 18-64 With Other Insurance Coverage at Dialysis Initiation (Days 2-365 of Dialysis Analysis), 2006-2016 **eFigure 3.** Annual Rates of Medicare Enrollment One Year After Dialysis Initiation, by Timing of Medicare Enrollment Decision (n=335,157)
