# Supplementary material for: Rates of Medicare Enrollment Among Dialysis Patients After Implementation of Medicare Payment Reform and the Affordable Care Act Marketplace
Source: JAMA Netw Open. 2022 Sep 20;5(9):e2232118. doi: 10.1001/jamanetworkopen.2022.32118 (PMC9490494; doi:10.1001/jamanetworkopen.2022.32118)
Supplement: Supplement. — eFigure 1. Patient Sampling Frame eFigure 2. Sample: Patients With Incident End-Stage Kidney Disease, by Insurance Status at Dialysis Initiation 2006-2016 eTable. Characteristics of Patients Age 18-64 With Other Insurance Coverage at Dialysis Initiation (Days 2-365 of Dialysis Analysis), 2006-2016 eFigure 3. Annual Rates of Medicare Enrollment One Year After Dialysis Initiation, by Timing of Medicare Enrollment Decision (n=335,157) [file jamanetwopen-e2232118-s001.pdf]

Supplemental Online Content

Wang V, Zepel L, Hammill BG, Hoffman A, Sloan CE, Maciejewski ML. Rates of Medicare enrollment among dialysis patients after implementation of Medicare payment reform and the Affordable Care Act Marketplace. *JAMA Netw Open*. 2022;5(9):e2232118. doi:10.1001/jamanetworkopen.2022.32118

eFigure 1. Patient Sampling Frame
eFigure 2. Sample: Patients With Incident End-Stage Kidney Disease, by Insurance Status at Dialysis Initiation 2006-2016
eTable. Characteristics of Patients Age 18-64 With Other Insurance Coverage at Dialysis Initiation (Days 2-365 of Dialysis Analysis), 2006-2016
eFigure 3. Annual Rates of Medicare Enrollment One Year After Dialysis Initiation, by Timing of Medicare Enrollment Decision (n=335,157)

This supplemental material has been provided by the authors to give readers additional information about their work.

© 2022 Wang V et al. *JAMA Network Open.*

eFigure 1. Patient Sampling Frame

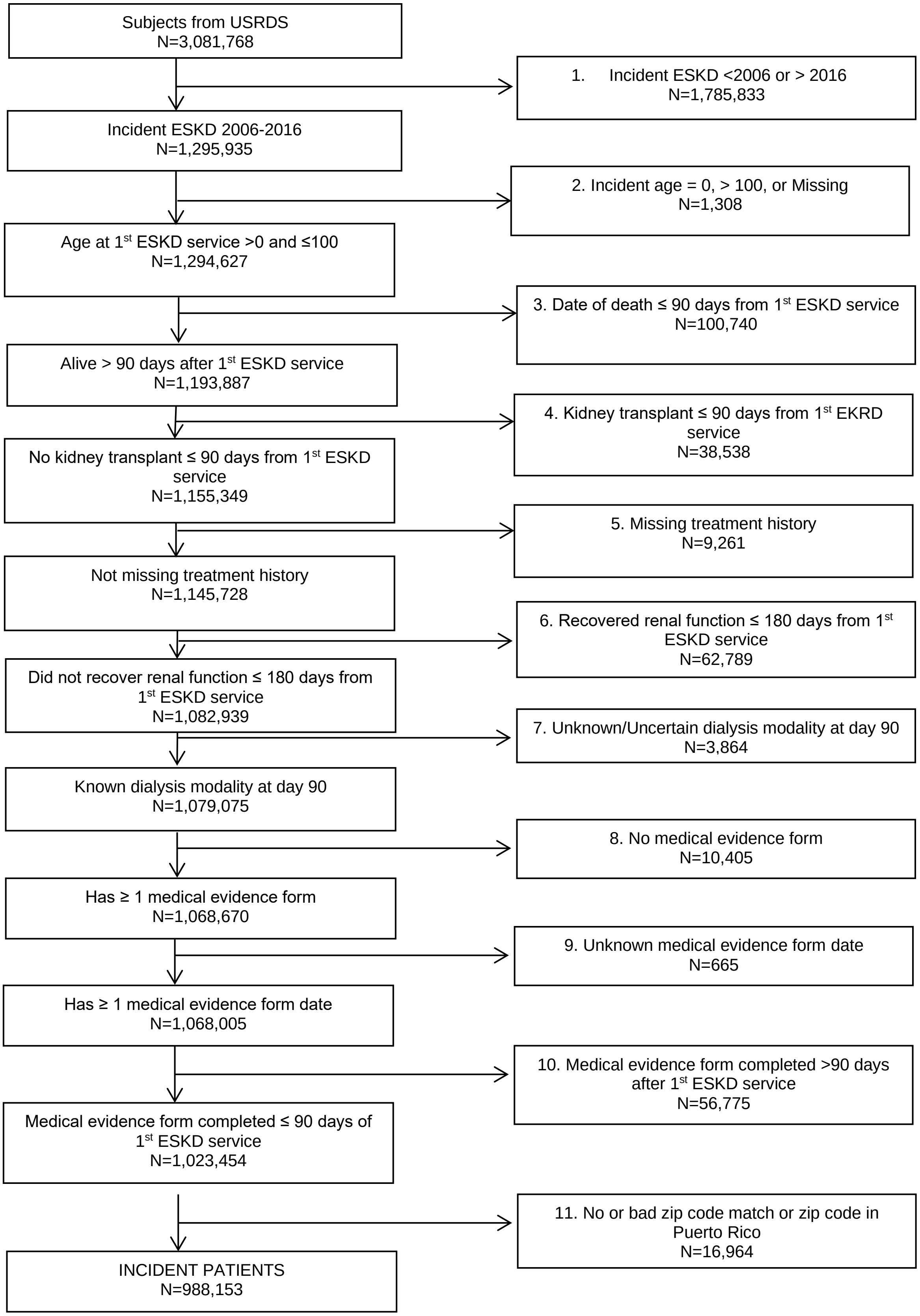

© 2022 Wang V et al. *JAMA Network Open.*

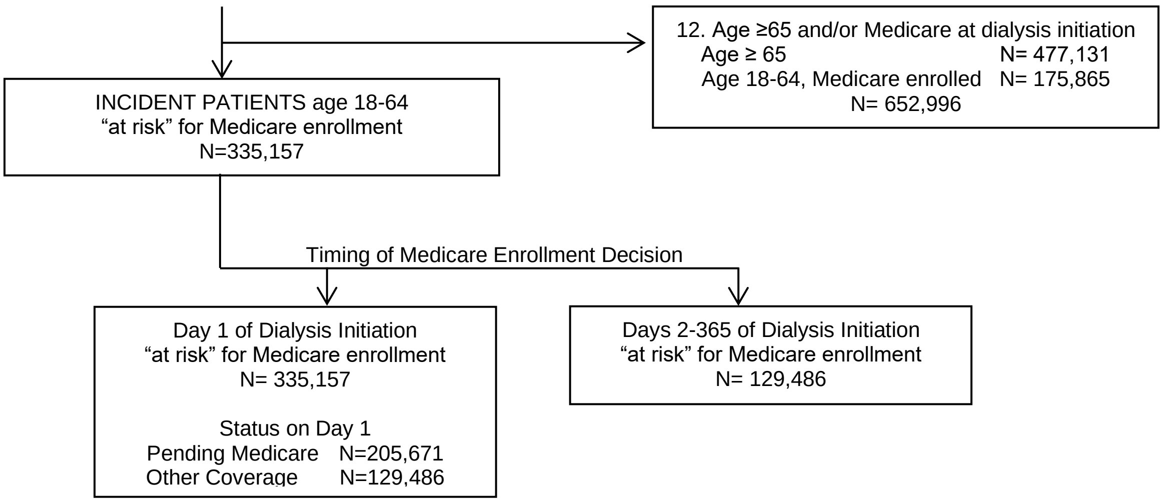

© 2022 Wang V et al. *JAMA Network Open.*

eFigure 2. Sample: Patients With Incident End-Stage Kidney Disease, by Insurance Status at Dialysis Initiation 2006-2016

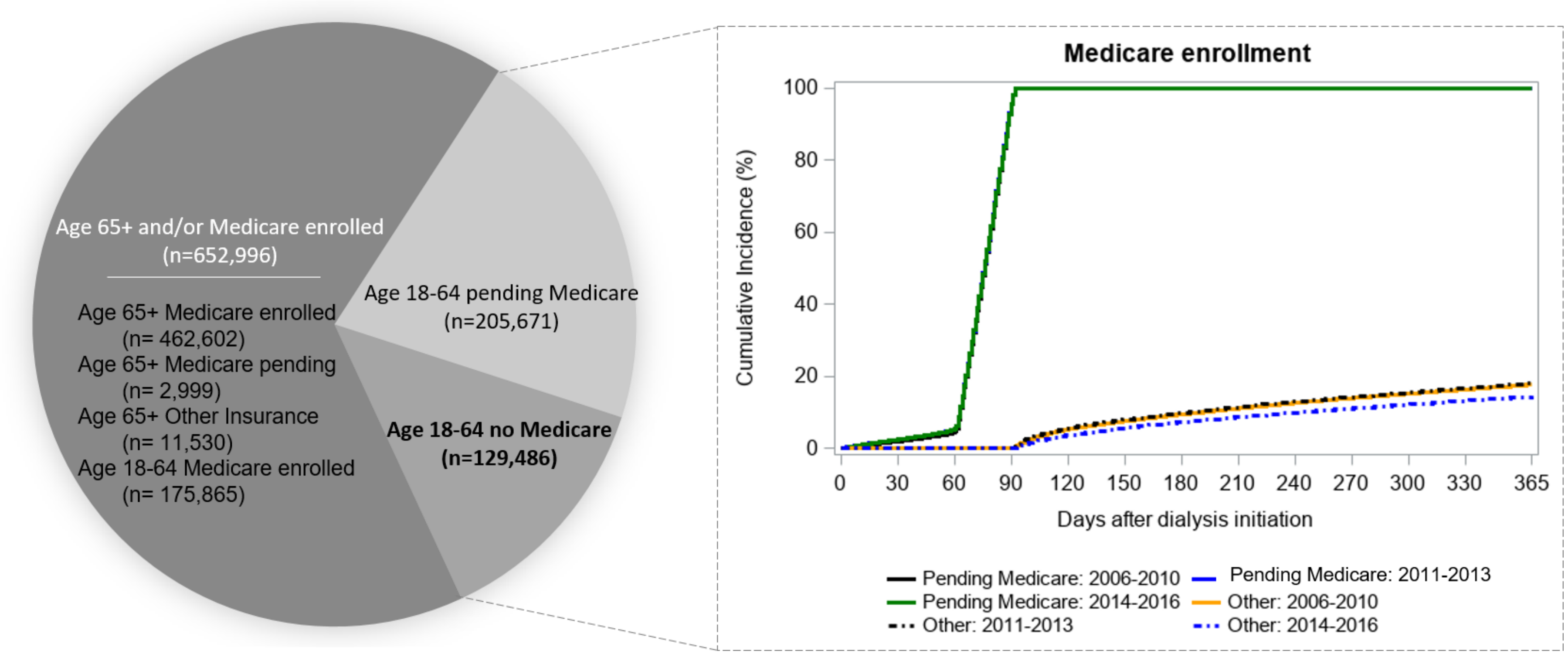

Note: Lines for all 3 time periods of those Pending Medicare at dialysis initiation overlap because those with Pending Medicare undergo a 90-day waiting period before enrollment is activated. By the end of the 90 days, these patients are actively enrolled in Medicare.

© 2022 Wang V et al. *JAMA Network Open.*

eTable. Characteristics of Patients Age 18-64 With Other Insurance Coverage at Dialysis Initiation (Days 2-365 of Dialysis Analysis), 2006-2016

	**OVERALL (n= 129,486)**	**2006-2010 (n=49,466)**	**2011-2013 (n=34,091)**	**2014-2016 (n=45,929)**
**Patient Characteristics, at Dialysis Initiation**				
Age (Mean, SD)	49.8 (11.1)	49.7 (11.0)	49.9 (11.1)	49.9 (11.1)
18-44	35,739 (27.6)	13,707 (27.7)	9,236 (27.1)	12,796 (27.9)
45-54	39,160 (30.2)	15,328 (31.0)	10,273 (30.1)	13,559 (29.5)
55-64	54,587 (42.2)	20,431 (41.3)	14,582 (42.8)	19,574 (42.6)
Male (N, %)	74,345 (57.4)	27,429 (55.5)	19,766 (58.0)	27,150 (59.1)
Race & ethnicity (N, %)				
White	71,366 (55.1)	26,114 (52.8)	19,000 (55.7)	26,252 (57.2)
Black	46,869 (36.2)	19,139 (38.7)	12,238 (35.9)	15,492 (33.7)
Other	11,251 (8.7)	4,213 (8.5)	2,853 (8.4)	4,185 (9.1)
Hispanic Ethnicity (N, %)	24,591 (19.0)	9,455 (19.1)	6,609 (19.4)	8,527 (18.6)
Employed full or part-time (N, %)	32,411 (25.0)	11,757 (23.8)	8,188 (24.0)	12,466 (27.1)
Urban residential status (N, %)	112,668 (87.2)	43,507 (88.1)	29,652 (87.2)	39,509 (86.3)
Region (N, %)				
South	44,069 (34.0)	15,813 (32.0)	11,437 (33.5)	16,819 (36.6)
Midwest	25,992 (20.1)	10,336 (20.9)	6,790 (19.9)	8,866 (19.3)
Northeast	26,507 (20.5)	10,653 (21.5)	7,190 (21.1)	8,664 (18.9)
West	32,918 (25.4)	12,664 (25.6)	8,674 (25.4)	11,580 (25.2)
Dialysis modality (N, %)				
In-Center Hemodialysis	115,274 (89.0)	45,318 (91.6)	30,290 (88.9)	39,666 (86.4)
Home Hemodialysis	369 (0.3)	113 (0.2)	123 (0.4)	133 (0.3)
Peritoneal Dialysis	13,843 (10.7)	4,035 (8.2)	3,678 (10.8)	6,130 (13.3)
Cause of ESKD (N, %)				
Diabetes	59,192 (45.7)	22,292 (45.1)	15,410 (45.2)	21,490 (46.8)
Hypertension	34,008 (26.3)	12,132 (24.5)	9,221 (27.0)	12,655 (27.6)

© 2022 Wang V et al. *JAMA Network Open.*

	OVERALL (n= 129,486)	2006-2010 (n=49,466)	2011-2013 (n=34,091)	2014-2016 (n=45,929)
Glomerulonephritis	15,728 (12.1)	6,294 (12.7)	4,185 (12.3)	5,249 (11.4)
Other	17,082 (13.2)	6,909 (14.0)	4,334 (12.7)	5,839 (12.7)
Unknown	3,476 (2.7)	1,839 (3.7)	941 (2.8)	696 (1.5)
Comorbidities [a] (N, %)				
Hypertension	112,989 (87.3)	42,724 (86.4)	29,936 (87.8)	40,329 (87.8)
Diabetes	68,839 (53.2)	25,616 (51.8)	18,069 (53.0)	25,154 (54.8)
Congestive heart failure	27,155 (21.0)	11,304 (22.9)	6,972 (20.5)	8,879 (19.3)
Atherosclerotic heart disease	12,159 (9.4)	5,636 (11.4)	3,211 (9.4)	3,312 (7.2)
Peripheral vascular disease	9,250 (7.1)	4,065 (8.2)	2,384 (7.0)	2,801 (6.1)
Pre-ESKD nephrology care, (N, %)				
Yes	73,196 (56.5)	26,481 (53.5)	19,273 (56.5)	27,442 (59.7)
No	38,666 (29.9)	16,564 (33.5)	10,239 (30.0)	11,863 (25.8)
Unknown	17,624 (13.6)	6,421 (13.0)	4,579 (13.4)	6,624 (14.4)
BMI, (Mean, SD)	30.0 (8.5)	29.7 (8.6)	30.2 (8.6)	30.3 (8.4)
eGFR [b], mL/mn/1.73m2 (Mean, SD)	10.3 (11.3)	10.7 (12.3)	10.4 (11.1)	9.8 (10.2)
**Market characteristics (Hospital Service Area)**				
% Freestanding facilities (Mean, SD)	91.3 (16.9)	88.9 (19.0)	91.6 (16.6)	93.6 (14.1)
% For-profit owned (Mean, SD)	84.7 (22.9)	81.7 (25.0)	85.2 (22.5)	87.6 (20.4)
% Chain affiliated (Mean, SD)	86.0 (21.3)	82.5 (23.7)	85.6 (21.4)	89.9 (17.6)
% Urban facility location (Mean, SD)	89.1 (29.3)	90.0 (28.2)	89.2 (29.2)	88.1 (30.5)
Dialysis market competition[c] (Mean, SD)	37.1 (34.1)	35.7 (33.6)	37.2 (34.1)	38.7 (34.5)
% Urban general population (Mean, SD)	86.2 (19.2)	86.9 (18.9)	86.3 (19.2)	85.4 (19.6)
Per capita income (Median, IQR)	$42,235 ($36,300, $49,623)	$38,773 ($33,352, $45,113)	$42,897 ($37,263, $48,943)	$45,824 ($39,893, $53,805)

© 2022 Wang V et al. *JAMA Network Open.*

eFigure 3. Annual Rates of Medicare Enrollment One Year After Dialysis Initiation, by Timing of Medicare Enrollment Decision (n=335,157)

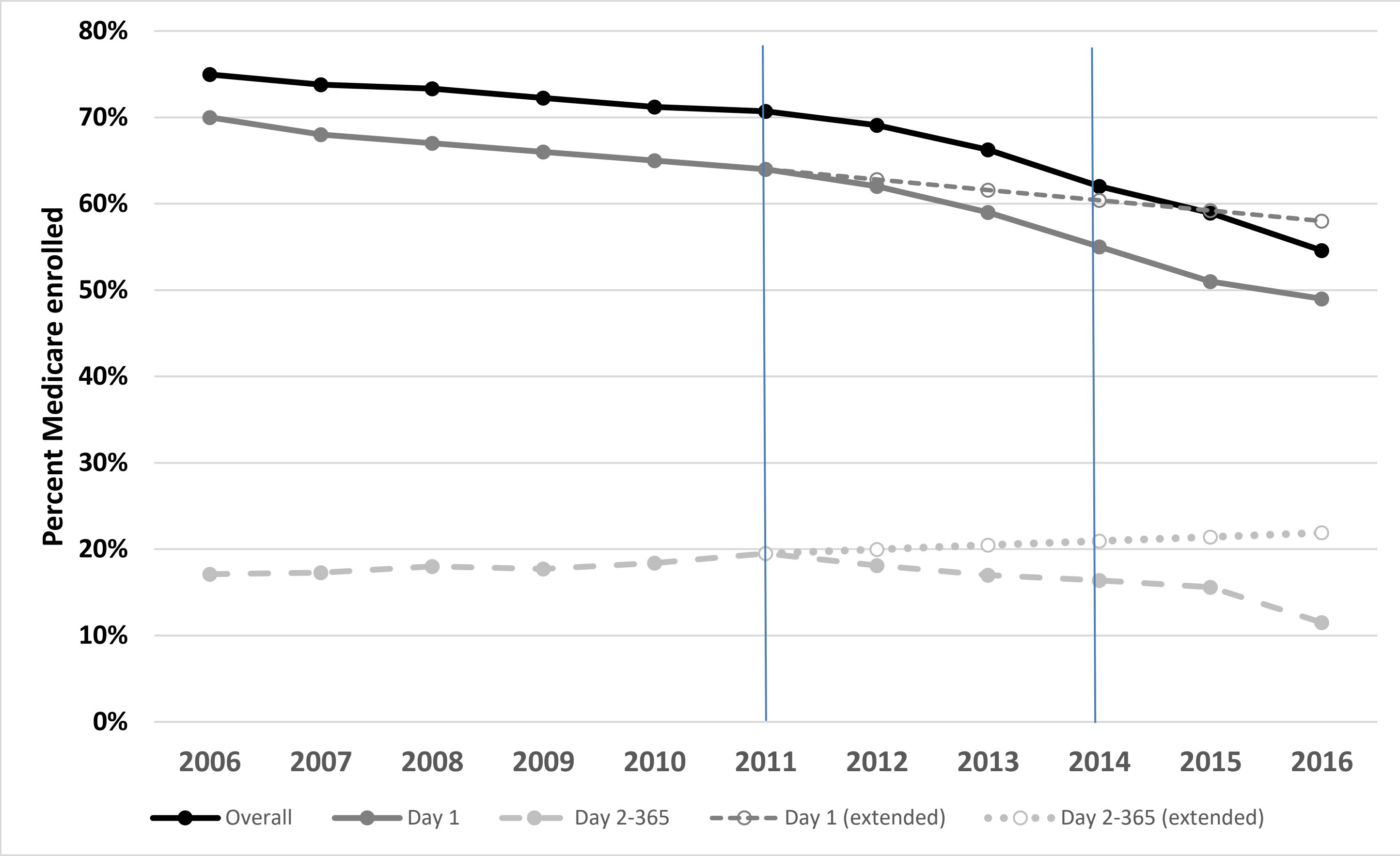

Note: Unadjusted annual rates of Medicare enrollment by the end of the first year of dialysis shows a general downward trend for patients' decision points for enrolling in Medicare: on *Day 1 of Dialysis Initiation* or *Days 2-365 of Dialysis Initiation*. This figure also extrapolates the 2006-2010 trends over the years 2011-2016 to that interpretation of our approach of modeling 3 policy periods is consistent with results that would generated from other models that specify time linearly in years (e.g., interrupted time series).

© 2022 Wang V et al. *JAMA Network Open.*
